# Temporal transcriptome analysis reveals several key pathways involve in cadmium stress response in *Nicotiana tabacum* L.

**DOI:** 10.3389/fpls.2023.1143349

**Published:** 2023-03-07

**Authors:** Chenyang Li, Yi Hong, Jinhao Sun, Guoping Wang, Huina Zhou, Liangtao Xu, Long Wang, Guoyun Xu

**Affiliations:** ^1^ China Tobacco Gene Research Center, Zhengzhou Tobacco Research Institute of China National Tobacco Corporation, Zhengzhou, China; ^2^ College of Biology, State Key Laboratory of Chemo/Biosensing and Chemometrics, and Hunan Province Key Laboratory of Plant Functional Genomics and Developmental Regulation, Hunan University, Changsha, China; ^3^ Technology Center, China Tobacco Jiangsu Industrial Co. Ltd., Nanjing, China; ^4^ Yuxi Zhongyan Tobacco Seed Co., Ltd., Yuxi, Yunnan, China

**Keywords:** plant transcriptomics, cadmium stress, temporal transcriptome, alternative splicing, *Nicotiana tabacum* L.

## Abstract

Tobacco has a strong cadmium (Cd) enrichment capacity, meaning that it can absorb large quantities from the environment, but too much Cd will cause damage to the plant. It is not yet clear how the plant can dynamically respond to Cd stress. Here, we performed a temporal transcriptome analysis of tobacco roots under Cd treatment from 0 to 48 h. The number of differentially expressed genes (DEGs) was found to change significantly at 3 h of Cd treatment, which we used to define the early and middle stages of the Cd stress response. The gene ontology (GO) term analysis indicates that genes related to photosynthesis and fatty acid synthesis were enriched during the early phases of the stress response, and in the middle phase biological process related to metal ion transport, DNA damage repair, and metabolism were enriched. It was also found that plants use precursor mRNA (pre-mRNA) processes to first resist Cd stress, and with the increasing of Cd treatment time, the overlapped genes number of DEGs and DAS increased, suggesting the transcriptional levels and post-transcriptional level might influence each other. This study allowed us to better understand how plants dynamically respond to cadmium stress at the transcriptional and post-transcriptional levels and provided a reference for the screening of Cd-tolerant genes in the future.

## 1 Introduction

In recent years, heavy metal pollution of farmland soil has become a prominent environmental problem (Yang et al., 2018; Qin et al., 2021). China alone loses roughly 10 million tons of grain every year due to this heavy metal pollution (Shibao et al., 2019). According to the national survey bulletin on soil pollution, more than 19.4% of the cultivated soil was contaminated, with Cd-contaminated soil accounting for 7.0% (Yang et al., 2022). The accumulation of high levels of cadmium in a plant can result in significant toxicity, leading to a range of disruptions that can damage the plant. These disruptions may include impacts on cell division, decreased growth rate, disruption of photosynthesis, alteration of plant enzyme activity and membrane integrity, and an increase in lipid peroxidation (Haider et al., 2021).

As a very important cash economic in China and the world, the Cd enrichment capacity of tobacco (Nicotiana tabacum) is of great interest. Excessive accumulation of Cd reduces the quality and yield of tobacco. At present, research on the mechanisms of how Cd affects tobacco growth and development has made some processes, however, most of this research focuses on the antioxidant effects of Cd on tobacco (Borgo et al., 2022). There are very few studies analyzing how tobacco responds to Cd stress and which genes participate in Cd response at pre-mRNA and mRNA levels (Fu et al., 2019). In recent years, the continuous development of high-throughput sequencing technology has increased the ability to study how plants respond to heavy metal pollution. Temporal transcriptome sequencing in particular has proven to be an effective way to study the response of plants to external environmental signals. This method could dynamically monitor plant responses to different stages of stimuli exposure at the gene expression level. For example, it has previously been used to reveal how wheat can coordinate the expression of homologous genes to cope with various environmental constraints at the genome-wide level (Hao et al., 2021); how candidate genes regulate flower opening and closing in Iris, leading to an improved understanding of the regulatory network of flowering (Liu et al., 2022).

Alternative splicing (AS) is a typical post-transcriptional regulation. It is a molecular mechanism that produces multiple mRNA transcripts from a single gene locus *via* the alternative selection of splicing sites during pre-mRNA processing (Lee and Rio, 2015). Studies have shown that AS plays an important role in plant responses to a variety of abiotic stresses, such as salt (Jian et al., 2022), droughts (Ding et al., 2022), and temperature stress (Weng et al., 2021), but there are still few studies on the response to Cd stress. Therefore, it is of great significance to understand the mechanism of plant response to Cd by using technologies such as differential expression genes (DEGs) and alternative splicing analysis.

In this study, we analyzed the response of tobacco roots to Cd stress by using a temporal transcriptome analysis. It was found that the number of DEGs increase significantly after 3 hours of Cd treatment. In addition, the main signal pathways were found to differ at various time points of the Cd stress response and plants employed pre-mRNA process to initially resist Cd stress. Therefore, this study provides data to support a dynamic understanding of plant Cd stress responses at transcriptional and post-transcriptional levels.

## 2 Materials and methods

### 2.1 Plant materials and cadmium stress treatment

The experimental material was the roots of tobacco cukltivar K326, which was bred by Northup King Seed Company by crossing McNair30 and NC95. Tobacco seeds were germinated in sterile, moist vermiculite. Uniformly healthy 5-leaf stage seedlings were transplanted into 1/2 MS medium (100 mL pyridoxine flask containing 50 mL Murashige and Skoog medium liquid medium, with vitamins and sugar), and containers were randomly placed in a greenhouse under natural light irradiation at a controlled greenhouse temperature of 25 ± 3°C. Plants were treated with 50 μM CdCl_2_ for 5 min, 15 min, 30 min, 1 h, 3 h, 6 h, 12 h, 24 h, and 48 h. At the end of the 48 h Cd treatment, tobacco roots, rinsed three times with deionized water and dried, and stored at -80°C. Samples without Cd treatment were labeled as T0 and samples under Cd treatment were labeled as T1-T9 depending on the treatment.

### 2.2 RNA-seq library construction

Samples from the different treatments were powdered in liquid nitrogen to extract RNA, and RNA-seq library was constructed as described previously (Wang et al., 2020). To perform RNA-seq, total RNA was first extracted from cells using a TRIzol reagent and treated with DNase to eliminate any genomic DNA contamination. The mRNA was then selectively captured using Oligo(dT) magnetic beads and fragmented using an interrupt reagent in a Thermomixer (Eppendorf, Hamburg, Germany). cDNA synthesis was carried out using the fragmented mRNA as template. Double-stranded cDNA was generated using a double-stranded cDNA synthesis reaction system and purified using a purification kit. The purified cDNA was then subjected to cohesive end repair, followed by the addition of an “A” base to the 3’ end and ligation of a linker. cDNA fragments of the desired size were selected, and PCR amplification was performed. The resulting libraries were sequenced using paired-end sequencing on an Illumina Hiseq4000 (BGI, Shenzhen, China) following the manufacturer’s protocol. In this study, thirty RNA libraries were generated, including three control libraries and twenty-seven treatment libraries, to investigate the effect of treatment on gene expression.

### 2.3 Bioinformatics analysis

Raw reads were analyzed using FastQC (https://www.bioinformatics.babraham.ac.uk/projects/fastqc/) prior to assembly. Trimmomatic v0.36 (http://usadellab.org/cms/?page=trimmomatic), parameter “CROP:150 ILLUMINACLIP : TruSeq3-PE-2.fa:2:30:10:8:true LEADING:3 TRAILING:3 SLIDINGWINDOW:4:15 MINLEN:4” is used to remove low quality sequences. Cutadapt (Martin, 2011) software was used to remove low quality reads, including those with sequencing adapters, sequencing primers and nucleotides with mass fractions below 20. At the same time, Q20, Q30 and GC content were also calculated for clean data. All downstream analyses were based on high quality clean data. The raw sequence data was submitted to the NCBI database under the registration number PRJNA93401.

Reference genome and gene model annotation files were downloaded from the Nicotiana tabacum K326 Flue-cured (https://solgenomics.net/organism/Nicotiana_tabacum/genome). The index of the reference genome was built using Hisat2 (v2.2.1) (Kim et al., 2019) and paired-end clean reads were aligned to the reference genome using Hisat2 (v2.2.1). Gene expression analyses were constructed as described previously (Wang et al., 2020). We used DEGseq2 R package (1.36.0) (Love et al., 2014) and using the normalization method of quantiles with fold change ≥2, and *q-value* < 0.05 as the threshold for determining whether the gene was differentially expressed to obtain DEGs, normalization of data using Z-score.

Alternative splicing events were identified using rMATS v4.1.1 (Shen et al., 2014) with parameters “-t paired –len 150 –c 0.0001 –analysis U –libType fr-firststrand –novelSS 1” and with the updated GTF file. Five types of AS events were then classified, including ES, A5SS, A3SS, MXE, and IR. Inclusion level (ψ) was a quantitative measurement of AS. With exon skipping as an example, inclusion level is estimated by the proportion of exon-exon junction counts supporting the exon-inclusion isoform. Inclusion level can be applied to all AS categories (exon skipping, intron retention, alternative 5′/3′ splice site, mutually exclusive exons), with minor changes made when defining “inclusion”. Parameters FDR < 0.05 and ΔPSI ≥ 0.1 were used to identify differential alternate splicing (DAS) events between the control and cadmium treatment groups.

### 2.4 Gene annotation and metabolic pathway analysis

Gene Ontology (GO) enrichment analysis of differentially expressed genes was implemented by the clusterProfiler (4.4.4) R package (Wu et al., 2021). GO terms with corrected *p-value* less than 0.05 were considered significantly enriched by differentially expressed genes. The clusterProfiler (4.4.4) R package was used to test the statistical enrichment analysis of differentially expressed genes in the KEGG pathway.

## 3 Results

### 3.1 Response of genes expression to cadmium stress in tobacco roots

**Figure 1:**
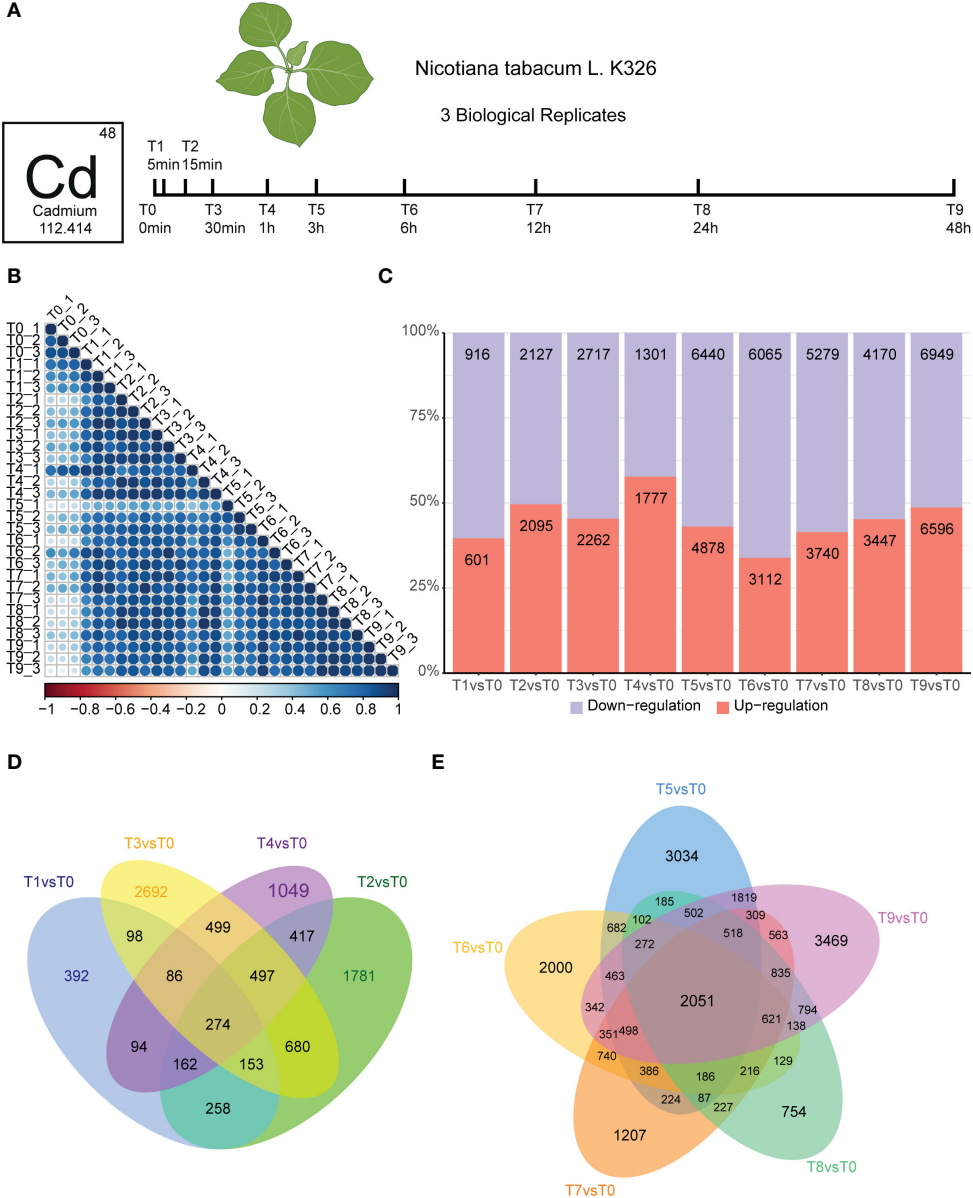
Experiment overview and effect of Cd stress response in tobacco root. **(A)**.Seedlings were incubated in a growth chamber until they grew to the five-leaf stage, then the roots of seedings were treated with 50 μM CdCl_2_ for different times. Three biological replicates of *Nicotiana tabacum* ecotype K326 were harvested. **(B)**. Heatmap depicting pairwise Pearson correlation of gene expression values of all samples. **(C)**. Bar graph showing the total number of differentially upregulated (orange) and downregulated (blue) genes in T1vsT0, T2vsT0, T3vsT0, T4vsT0, T5vsT0, T6vsT0, T7vsT0, T8vsT0, and T9vsT0 samples. **(D)**. Venn diagram showing common and unique genes in T1vsT0, T2vsT0, T3vsT0, and T4vsT0 samples. The number of genes intersecting all time points was 274. **(E)**. Venn diagrams showing differential gene statistics for T5vsT0, T6vsT0, T7vsT0, T8vsT0, and T9vsT0 samples. The number of genes intersecting all time points was 2,051.

To analyze how tobacco roots dynamically respond to cadmium stress, we treated 5-leaf stage *Nicotiana tobacco* with 50 μM cadmium ions and collected samples at different time points (0, 5 min, 30 min, 1 h, 3 h, 6 h, 24 h, and 48 h) for RNA-seq (**Figure 1A**). We conducted three biological replicates per sample. A time-series differential expression analyses were performed to explore the global temporal patterns of transcriptomic changes. We generated at least 10 Gb of clean bases for each sample, and more than 85% of the sequencing reads could be uniquely mapped to the *N. tabacum* K326 genome, indicating the high quality of the RNA-seq data (**Table S1**). Correlation analyses were used to estimate the relationships between the 30 transcriptome samples. Our results showed that all replicated data was generally well correlated within the same time point (T1-T9; **Figure 1B**).

We then identified the DEGs occurring in the root of K326 following exposure to Cd stress treatment. We monitored how these changes occurred over different time and compared them with the untreated control (T0). Our results identified a total of 64,472 DEGs by using the edgeR package with *q-value* < 0.05, fold change ≥2. Compared with the T0 group, within 5 minutes there were 601 up-regulated genes in the Cd stress-treated group, while 916 DEGs were down-regulated in the T1 vs T0 Cd stress-treated group (**Figure 1B**). The number of DEGs increased rapidly, as T1 vs T0 group contained almost 1/3 of those in the T2 vs T0 group (**Figure 1B**). This degree of variation suggests that the mRNA response strategy has achieved a certain scale after 15 min of Cd treatment. The number of DEGs responding to Cd stress increased significantly after 3 h compared to 1 h (**Figure 1C**), and the highest number of DEGs were detected in the 48 h samples. Based on these results, the treatment time points were divided into early (T0 to T4) and mid-term (T5 to T9) responses to Cd stress. Additionally, we detected 274 overlapped genes in the Venny analysis of the early stage of Cd stress, which reached 5.5-18% of the total DEGs (**Figure 1D**). We also detected 2,051 overlapped genes in the Venny analysis of the mid-term stage of Cd stress, which reached 15-22% of total DEGs (**Figure 1E**). In summary, the number of DEGs in the middle stages of Cd treatment was significantly increased compared with that in the early stage of treatment. The proportion of overlapping genes is significantly higher in the mid-stage compared to the early stage, indicating that more common genes are synergistically involved in the cadmium response in the mid-stage.

### 3.2 GO term and KEGG analysis in the early stage of Cd treatment

**Figure 2:**
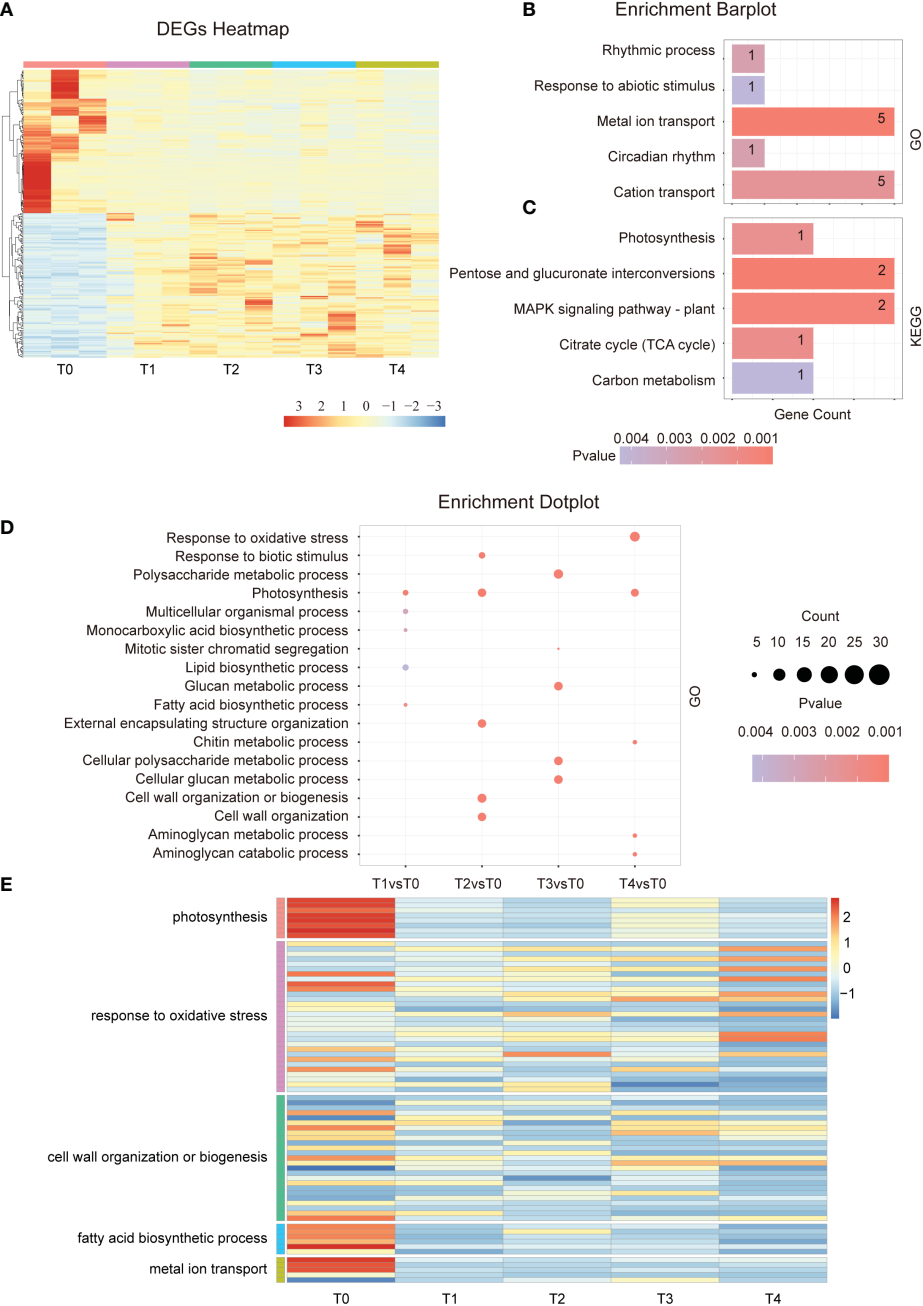
Differential gene expression in the early stage of Cd treatment. **(A)**. Hierarchical clustering heat map showing the gene expressions of a total of 15 samples at each time point during the early phase of Cd stress treatment (T0-T4). **(B)**. GO term enrichment analysis of common DEGs after cadmium treatment (T1vsT0, T2vsT0, T3vsT0, and T4vsT0). The top 5 entries were selected for shown. **(C)**. KEGG analysis of common DEGs after cadmium treatment (T1vsT0, T2vsT0, T3vsT0, and T4vsT0). The top 5 entries were selected for statistical analysis. **(D)**. GO enrichment results of differentially expressed genes at each time point (T1-T4) in the early phase of the Cd stress response. The top 3 entries for each time significance were selected for statistical analysis. **(E)**. The heat map shows the gene expression in photosynthesis, response to oxidative stress, cell wall organization or biogenesis, fatty acid biosynthetic process, and metal ion transport at the early stage of cadmium stress (T0-T4).

To investigate the response mechanisms in plants at the early stage of Cd stress treatment, we analyzed the DEGs from T0 to T4. The DEGs heatmap show that the number of up- and down-regulated genes from the 274 overlapped genes is similar between T1-T4 samples (**Figure 2A**). During our experiment, a gene ontology enrichment analysis revealed that the 274 overlapped genes were mainly involved in the rhythmic process, response to abiotic stimulus, metal ion transport, circadian rhythm, and cation transport et al. (**Figure 2B**). Circadian rhythms are essential for normal gene expression, indicating that Cd treatment could induce a disorder in the expression of rhythm-related genes. KEGG analysis indicated that in response to the early stage of Cd exposure, the overlapped DEGs enriched photosynthesis, MAPK signaling pathway, TCA cycle, and carbon metabolism (**Figure 2C**). These results demonstrate that Cd stress affects the photosynthesis of plants, leading to a decline in the overall photosynthetic rate. Moreover, other corresponding energy metabolism processes must also be affected. Therefore, the continuously changing, overlapping genes are mostly related to energy metabolism, metal ion transport, and abiotic stimulus response.

Next, we analyzed the GO term of all multiple specific response pathways at each time point in the early stage. The results showed that the photosynthesis, lipid biosynthetic process, and fatty acid biosynthetic processes were enriched after 5 minutes of Cd treatment (**Figures 2D, E**). After 15 minutes of Cd treatment, the pathways related to cell wall organization and response to biotic stimulus were affected, and after an hour, the plant began to respond to oxidative stress (**Figures 2D, E**). A similar conclusion was found by analyzing the KEGG pathway which affected the photosynthesis and fatty acid elongation after just 5 minutes of treatment. After 15 min of treatment, plant hormone signal transduction, MAPK signaling pathway, and phenylpropanoid biosynthesis were also enriched (**Figure S1A**). In summary, the main pathways involved in Cd stress response occur at different stages. Several signal pathways are continuously affected after Cd treatment such as photosynthesis and lipid biosynthesis. However, more signal pathways have a spatio-temporal specific response.

### 3.3 GO term and KEGG analysis in the middle stage of Cd treatment

**Figure 3:**
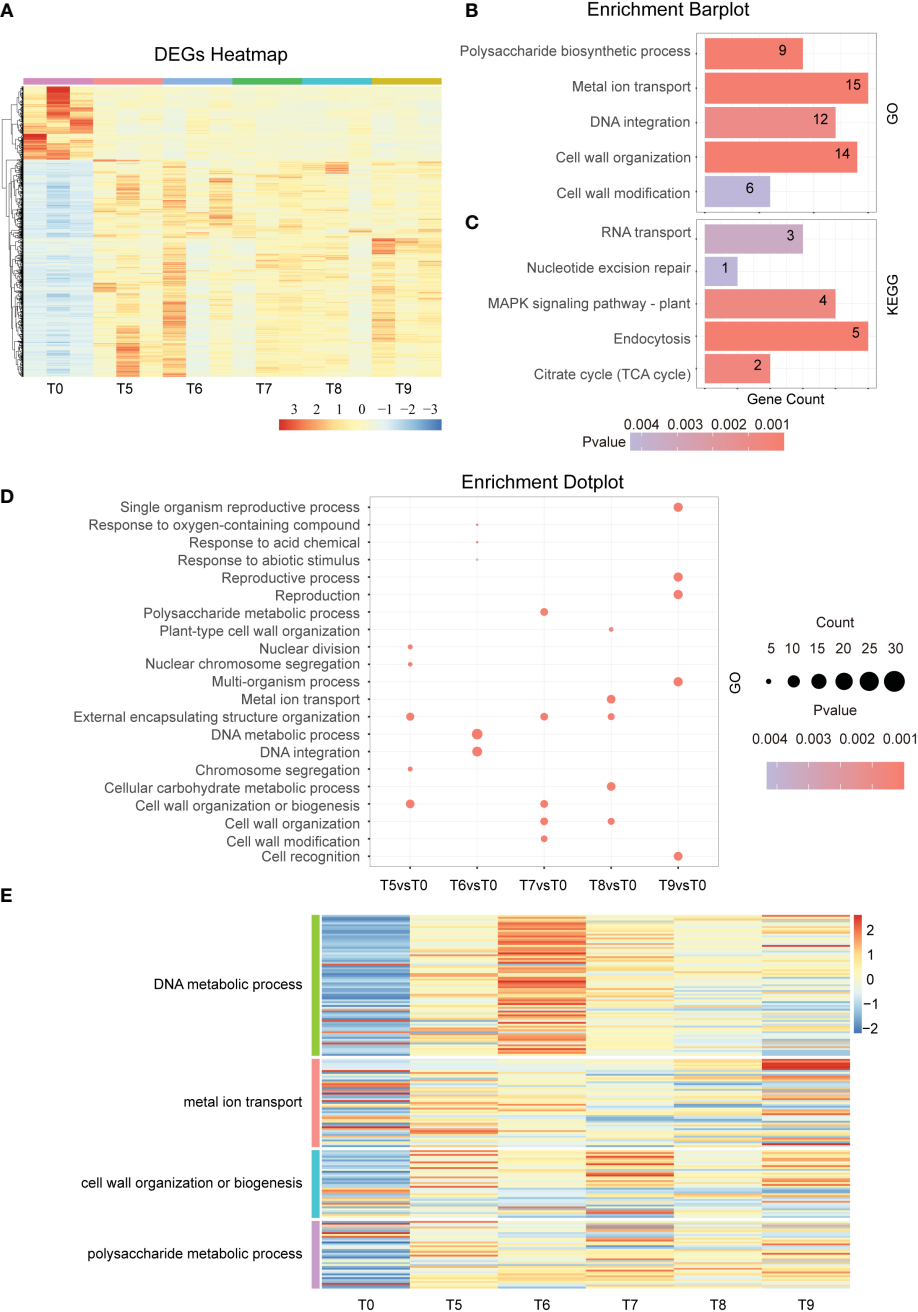
Differential gene expression in the middle stage of Cd treatment. **(A)**. Hierarchical clustering heat map showing the gene expression of a total of 18 samples at each time point during the middle phase of Cd stress treatment (T0 and T5-T9). **(B)**. GO term enrichment analysis of common DEGs after Cd treatment (T5vsT0, T6vsT0, T7vsT0, T8vsT0, and T9vsT0). The top 5 entries for each time significance were selected for statistical analysis. **(C)**. KEGG analysis of common DEGs after cadmium treatment (T5vsT0, T6vsT0, T7vsT0, T8vsT0, and T9vsT0). The top 5 entries for each time significance were selected for statistical analysis. **(D)**. GO enrichment results of differentially expressed genes at each time point (T5-T9) in the middle phase of the Cd stress response. The top 3 entries for each time significance were selected for statistical analysis. **(E)**. The heat map shows the gene expression in DNA metabolic process, metal ion transport, cell wall organization or biogenesis, and polysaccharide metabolic process at the middle stage of cadmium stress (T5-T9).

To investigate the response mechanisms at the middle stage of the Cd stress response, we analyzed the RNA-seq transcriptomic data of tobacco under Cd treatment from T5 to T9. The results displayed in the DEGs heatmap showed that the number of up-regulated genes is about three times that of down-regulated genes in the 2,051 overlapped genes (**Figure 3A**). Among these genes, GO enrichment analysis showed that cell wall modification, DNA integration, cell wall organization, polysaccharide biosynthetic process, and metal ion transport were enriched (**Figure 3B**). Likewise, the KEGG analysis showed that the RNA transport, nucleotide excision repair, MAPK signaling pathway, and endocytosis pathway were also enriched (**Figure 3C**). Moreover, the GO term analysis revealed that nuclear division, chromosome segregation, and cell wall organization or biogenesis were enriched at T5 vs T0. The GO term related to DNA metabolic processes and DNA integration were enriched in T6 vs T0 (**Figures 3D, E**). Similarly, signal pathways changed as time continued, especially at 48h of Cd treatment where it was noted that the main enrichment pathway becomes more involved in reproductive processes and cell recognition (**Figures 3D, E**). This led to a significant difference between the main KEGG pathways after 48 h and all other time points (**Figure S1B**). These results indicate that cell walls are disrupted by continuous Cd stress and that the early responses to exogenous Cd and abiotic stimuli are gradually converted into pathways related to DNA damage and cell well repair.

### 3.4 Transcriptional dynamics of cadmium stress response in tobacco roots

**Figure 4:**
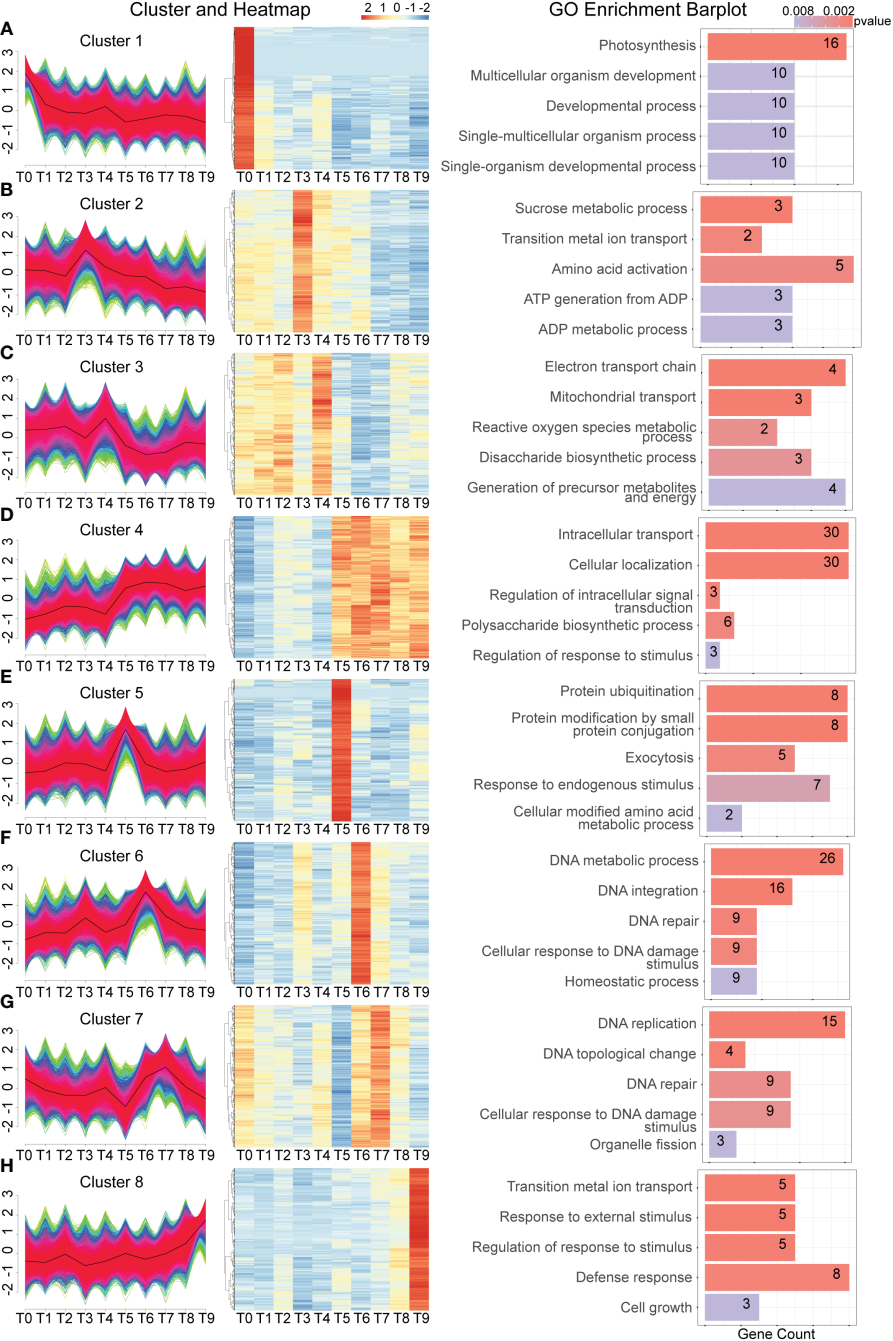
Time-course analysis of dynamic gene expression changes during Cd treatment. **(A–H)**. From left to right, gene expression is divided into 8 significant clusters based on the similarity of gene expression in each sample. The right side is a heat map of the expression of the top 1,000 genes most relevant to each cluster in each sample, graphically showing the top 5 significant GO terms.

To analyze gene expression patterns, a time-course analysis was conducted by clustering all genes from different time points to investigate their expression dynamics. Thousands of Cd-response genes were classified into 8 different expression patterns based on their response to the Cd treatment (**Figures 4A–H**). The expression of genes in cluster 1 was the highest at T1 and gradually decreased over time. GO analysis indicated that these genes are involved in photosynthesis, multicellular organism development, and development processes. This indicates that Cd stress first destroys a plant’s photosynthesis which leads to an effect on plant growth. Genes in clusters 2 and 3 showed similar expression patterns (**Figures 4B, C**), exhibiting a rapid response to Cd stress at 30 min and 1h, respectively. The GO analysis indicates that these genes are involved in sucrose metabolic processes, amino acid activation, reactive oxygen species, the generation of precursor metabolites, and energy, meaning that a plant likely produces energy to resist Cd stress after exposure.

Genes in clusters 5 and 6 also followed this expression pattern. At 3 h after Cd treatment, both the protein ubiquitination and response to endogenous stimulus were enriched. GO analysis indicated that genes enriched DNA metabolic process, DNA integration, DNA repair, and the cellular response to DNA damage stimulus at 6 h (T6) following Cd treatment (**Figures 4D–F**). It has been reported that in response to Cd stress, many genes with repair functions become active to mend the damaged parts. A similar function was observed at the T7 time point, where many genes with repair functions became active due to the altered cell structure (**Figure 4G**). Due to the persistence of cadmium stress, genes related to cell growth and defense response continued to be active at the T9 time point (**Figure 4H**). Collectively, these expression patterns illustrate the relationship similarities and differences between the early and middle stages of the Cd stress response.

### 3.5 Identification of different alternative splicing events in response to Cd stress

**Figure 5:**
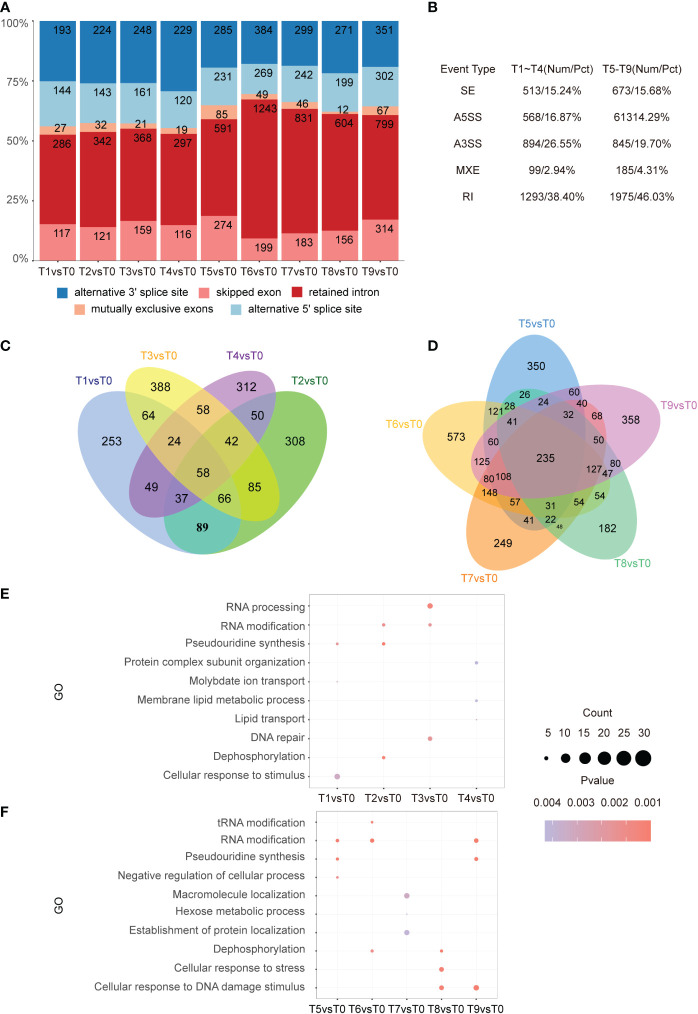
Distribution of AS types. **(A)**. Bar graph showing the number of AS events at each time point from T1 to T9. **(B)**. Five different types of AS events and their frequency. The first column is the AS event category, the second column is the corresponding early stage of the stress with its proportion, and the third column is the corresponding mid-stress event with its proportion. **(C, D)**. From left to right, the Venn diagram shows the DAS gene differences at each time point in the pre-response (T1-T4) and mid-response (T5-T9) periods to Cd stress. In the pre-response period, there were 58 intersecting genes. In the mid-response period, there were 235 intersecting genes. **(E, F)**. From top to bottom, the results of GO enrichment analysis of DAS genes in each time point in early (T1-T4), as well as mid-response (T5-T9) are shown. The top 3 entries for each time significance were selected for statistical analysis.

To identify the different alternative splicing (DAS) events that were sensitive to cadmium stress, AS analysis was performed using rMATS. A total of 3,367 DAS events from 1,883 genes were identified in tobacco roots. We found that similar to the sharp increase of DEGs in T5 vs. T0, the number of genes with AS increased significantly during this time point (**Figure 5A**). Among the DAS types between T1-T4, retained intron (RI) was the most abundant (38.40%), followed by alternative 3’SS (26.55%), alternative 5’SS (16.87%), exon skipping (15.24%), and mutually exclusive exons (2.94%) (**Figure 5B**). We identified 4,291 AS events from 3,519 genes over a total of five time periods from 3 h to 48 h. Among the AS types, RI was the most abundant (46.03%), followed by A3SS (19.70%), SE (15.68%), A5SS (14.29%), and MXE (4.31%). In addition, we identified 58 and 235 overlapped AS events in the early and middle stages of Cd stress, respectively (**Figures 5C, D**).

Similar enrichment analysis was performed for differential AS events in the early stage of cadmium stress treatment. It showed that cellular responses to stimulus were enriched in the T1 time point, which indicates that plants begin to respond to Cd stress by regulating the AS of stimulus-response genes 5 min after treatment. In addition, the AS events were also involved in RNA processing, RNA modification, DNA repair, and dephosphorylation processes in the early stage of Cd treatment (**Figure 5E**). Meanwhile, GO analysis showed enrichment in cellular responses to stress, DNA damage, and RNA modification in the T8 and T9 time points (**Figure 5F**). The same enrichment results are reflected in the KEGG pathway analysis, where the differential AS genes were mostly expressed in rhythm-related pathways and various amino acid synthesis pathways in the early stage of the stress response (**Figure S2A**). In the middle stage of the stress response, there was an enrichment of the mRNA surveillance pathway, N-Glycan biosynthesis, and spliceosome (**Figure S2B**).

To analyze the relationship between DEGs and DAS, we conducted a combined analysis. For the early stage (T0-T4) Cd stress response, DE and DAS genes differed greatly, with very few intersections. 77 intersecting genes were present in the middle phase (T5-T9). GO terms such as cellular response to stimulus, signal transduction, single organism signaling, and cell communication as well as some terms related to signaling and modifications were common to both DE and DAS genes. The two time points (T5 and T6) with the highest percentage of RI events were analyzed (**Figures S3A, B**). At 3 h of Cd stress treatment, the GO term showed gene enrichment in dephosphorylation which was associated with nucleotide anabolism (**Figure S3C**). After 6 h, the GO term showed genes associated with amino acid activation, polysaccharide synthesis, and tRNA anabolism (**Figure S3D**). In summary, we found that the response of AS to Cd stress is faster than that at transcriptional levels due to AS event that response to stimulus appears in the process of pre-mRNA processing within 5 min, and the transcriptional levels and post-transcriptional level might influence each other.

## 4 Discussion

To cope with the various environmental stresses that adversely affect normal plant growth and development, plants usually employ a variety of different strategies to respond to different durations of exposure (Chaturvedi et al., 2021). In this study, we chose to analyze the root response, since they are one of the main organs that sense environmental cadmium concentration and absorb it. To this end, we used a multi-time-point transcriptome analysis of tobacco from 5 min to 48 h of cadmium stress treatment. We observed changes in the number of differentially expressed genes in the root transcriptome at different stages of the treatment. A significant increase in the number of DEGs was observed in the T5 time point compared with T4 time point, and we used this as a boundary to classify 0 minutes to 1 hour as the early-stress response and 3 hours to 48 hours as the mid-stress response. Therefore, the effect of cadmium treatment on different genes expression varies at early and mid-stages.

Photosynthesis is an important component of plant metabolism and its integrity is crucial for normal plant growth and development (Dubey, 2018). Photosynthesis was affected after only 5 min of stress due to the massive translocation of metal cations into the plant. There were also many differentially expressed genes that were enriched, causing an effect on many functions related to photosynthesis. This is consistent with the reported toxic effect of the heavy metal Cd on plant photosynthesis (Dias et al., 2012). Changes in the photosynthetic capacity also affect primary carbon metabolism processes, which in turn causes an effect on the tricarboxylic acid cycle (Heyneke and Fernie, 2018).

In the early-response period, fatty acid synthesis-related pathways are enriched. Fatty acids play an important role in land plant responses to abiotic stress. They serve as reserves of carbon and energy and provide extracellular barrier components, and stress signal regulators (He and Ding, 2020). The plant cell wall is one of the most important physical factors for resisting abiotic stress, the accumulation of lignin or corkiness in the primary cell wall prevents the entry of foreign substances (Keegstra, 2010). It has been shown that cadmium causes some degree of damage to the cell wall in plants (Gutsch et al., 2018), and genes associated with cell wall repair are active under the continuation of the stress process. Cadmium can cause RNA single-strand breaks that line into DNA base modification products affecting DNA repair (Zhang et al., 2019). As cadmium continues to accumulate in the middle of the stress response, apoptosis and DNA damage occur, and in response, the genes related to DNA repair are enriched.

It has been shown that alternative splicing may be associated with transcriptional regulation in response to environmental stress in plant. This association is related to different species or different stress (Staiger and Brown, 2013). In rice (Dong et al., 2018), there was little overlap between DASs and DEGs under different mineral-deficient conditions, whereas there was a strong overlapping relationship between the two under drought stress in tea tree (Ding et al., 2020). In wheat samples heat-treated for one-hour, pre-mRNA processing was significantly associated with transcriptional regulation (Liu et al., 2018). Early in the stress response, differentially expressed genes rarely intersected with differentially AS genes. As the treatment time of cadmium stress prolonged, the overlapped genes number of DEGs and DAS increased gradually. One possibility is that Cd stress might affect the relationship between transcription and post-transcriptional level, which needs to be further explored in the future.

Additionally, we found that several receptor kinases are induced to express in response to stress, such as gene_84425, and receptor kinases are widely involved in the process of plant stress resistance (Zhao et al., 2021), therefore this kind of genes may be used.

## 5 Conclusions

In this study, we investigated the transcriptome of tobacco in response to Cd stress using RNA-seq. Based on the results, we identified stage-specific differences in tobacco during prolonged treatment and divided samples into two response periods. The early phase of the stress response was characterized by a large translocation of metallic cadmium ions which affected the rhythm-related pathways, while in the middle phase of the stress response there was a shift from receiving stimuli toward repairing damage. We found that plants tend to change and increase the number of splicing patterns in response to long-term Cd stress, and alternative splicing regulation of stimulus response occurs prior to and influences transcriptional regulation.

## Data availability statement

The datasets presented in this study can be found in online repositories. The names of the repository/repositories and accession number(s) can be found below: NCBI accession PRJNA913401.

## Author contributions

GX and LW conceived the project and designed research. CL and YH performed research. JS, HZ, GW, LX, and CL contributed new reagents/analytic tools. CL and LW wrote the paper. All authorscontributed to the article and approved the submitted version.
