# Supplementary material for: Temporal transcriptome analysis reveals several key pathways involve in cadmium stress response in Nicotiana tabacum L
Source: Front Plant Sci. 2023 Mar 7;14:1143349. doi: 10.3389/fpls.2023.1143349 (PMC10027936; doi:10.3389/fpls.2023.1143349)
Supplement: Supplementary file 1 [file DataSheet_1.docx]

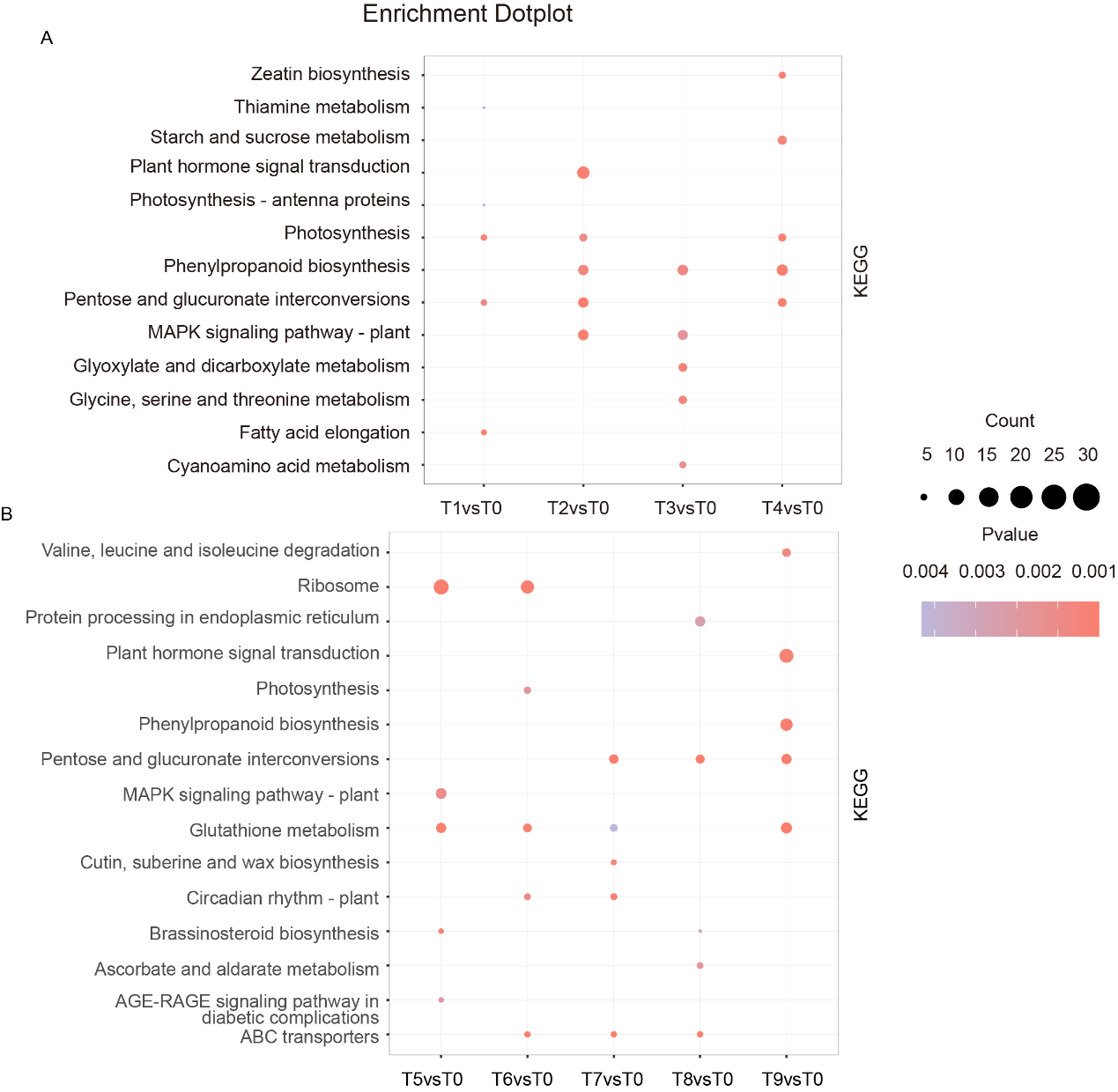


**Figure S1. KEGG results for the DEGs at each time point from T1 toT9**

A-B. From top to bottom, the KEGG enrichment results of differentially expressed genes at each time point in the early phase (T1-T4) and middle phase (T5-T9) of the Cd stress response. The top 3 entries for each time significance were selected for statistical analysis.


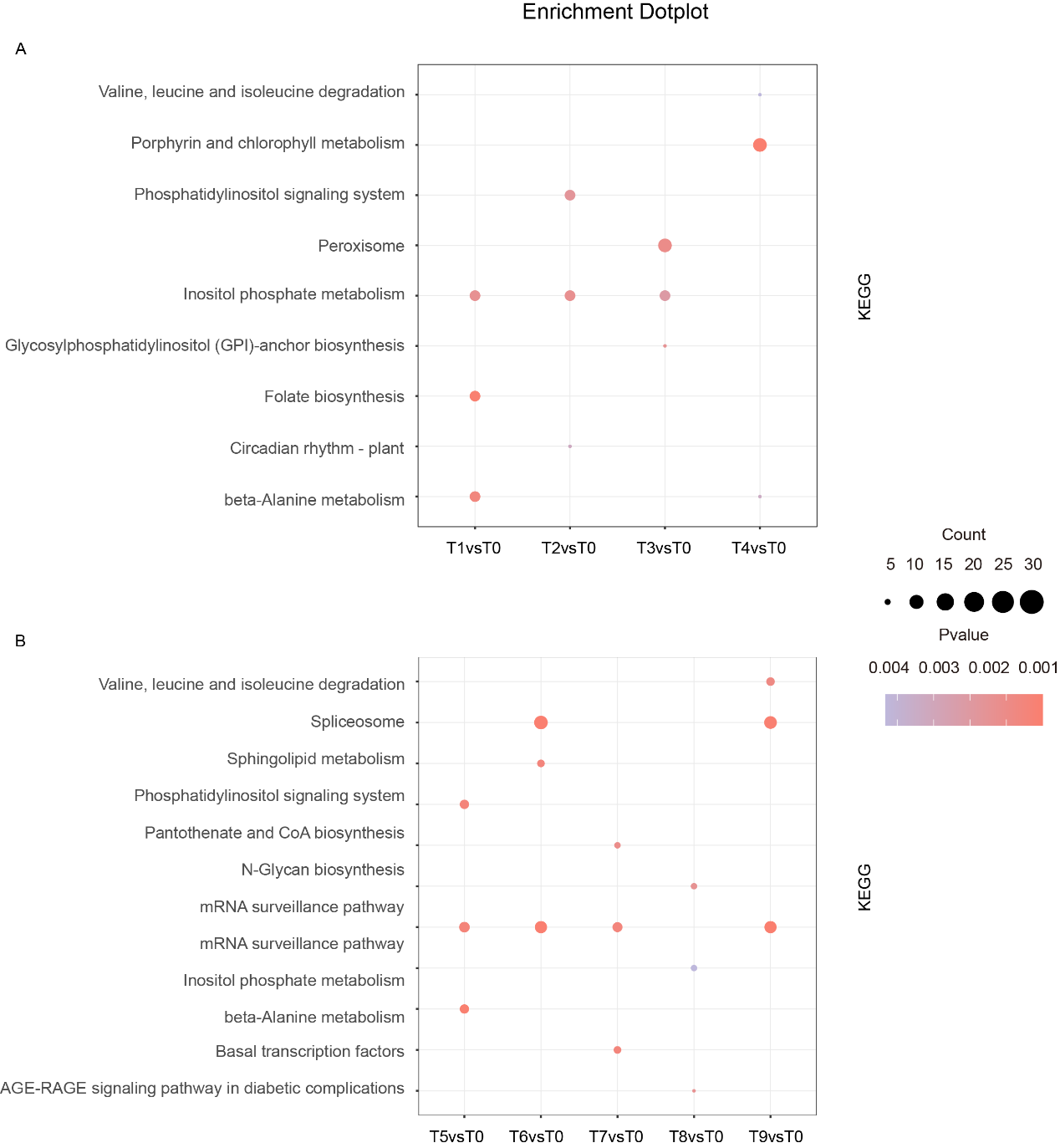


**Figure S2. KEGG results for the DAS genes at each time point from T1 toT9**

A KEGG results of DAS genes in the early stage of cadmium stress (T1-T4). The top 3 entries for each time significance were selected for the statistics.

B KEGG results of DAS genes in the middle period (T6-T9) of cadmium stress. The top 3 entries for each time significance were selected for the statistics.


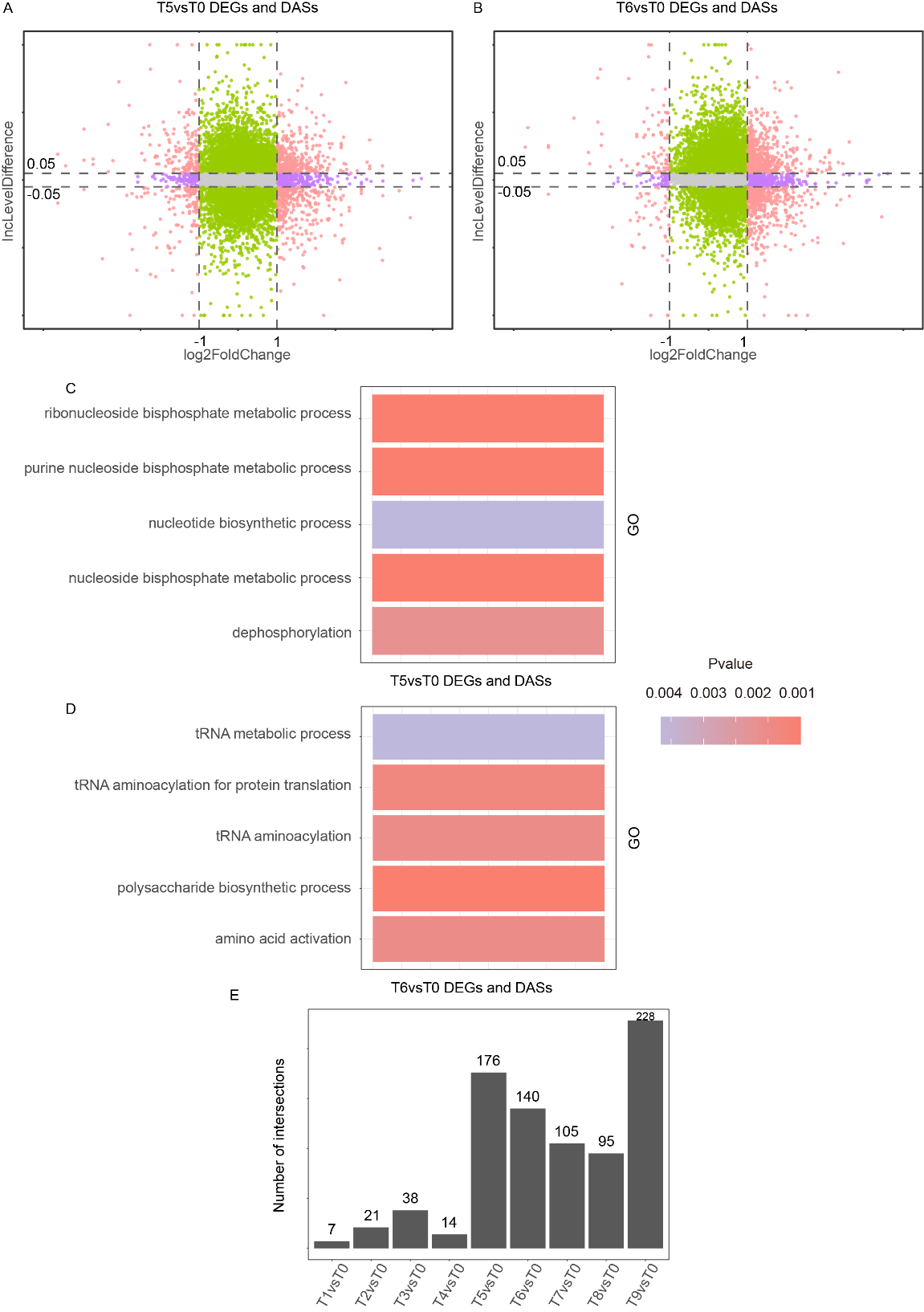


**Figure S3. Differentially expressed (DE) and DAS analyses of tobacco in response to Cd stress at 3 h and 6 h**

A-B. Association between DE and DAS in tobacco under Cd stress treatment for 3h and 6h. Variable shear event rate of change threshold of 0.05 and differential expression threshold of 1.

C-D. Go term enrichment analysis between DE and DAS at 3h and 6h. The top 5 entries for each time significance were selected for the statistics.

E. Number of DEGs intermingling with DAS genes under different times of cadmium treatment
